# Management of Recurrent Pregnancy-Related Pericarditis

**DOI:** 10.1155/2022/5791307

**Published:** 2022-03-22

**Authors:** Michel Ibrahim, Michael Fattouh, Alice Jacobs

**Affiliations:** ^1^Department of Medicine, Cardiovascular Division, Temple University Hospital, Philadelphia, PA, USA; ^2^Montefiore Medical Center, Bronx, NY, USA; ^3^Boston University Medical Center, Boston, MA, USA

## Abstract

The evidence on recurrent pregnancy-related pericarditis is limited, and management strategies are based on case reports and expert opinion. We describe a patient with myopericarditis complicated by cardiac tamponade presenting shortly after her first pregnancy, which was then complicated by refractory recurrent pericarditis. She was treated with standard first line therapies, such as NSAIDs, corticosteroids, and colchicine, and eventually initiated on the purine analog, azathioprine. Out of fear of teratogenicity, she self-discontinued her maintenance medications and thereafter, her course was complicated by a recurrent flare of pericarditis during a subsequent pregnancy. Our case illustrates the significant burden on our patient due to the incessant nature of her disease and on the providers due to the therapeutic dilemmas associated with family planning and pregnancy. Further data is required on this unique clinical scenario, and patient-centered management by a multidisciplinary team is critical.

## 1. Background

Acute pericarditis is among the most common pericardial diseases seen in pregnancy [1, 2], with recurrent disease being one of the most troublesome complications of pericarditis. The evidence on recurrent pregnancy-related pericarditis is limited, with the largest study on this topic consisting of a case series of 21 pregnancies [3]. As such, management strategies are based on case reports and expert opinion. However, in pregnant patients, the issue of pharmacotherapy is an important one, and recurrent pericarditis related to pregnancy poses a therapeutic dilemma. Our case illustrates a tailored multidisciplinary approach to the management of pregnancy-related idiopathic recurrent pericarditis (IRP), and we describe the current data surrounding this topic.

## 2. Case Presentation

**Figure 1:**
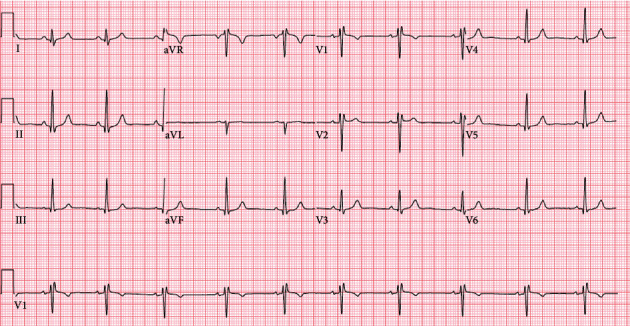
Normal sinus rhythm. RSR′ or QR pattern in V1 suggests right ventricular conduction delay.

**Figure 2:**
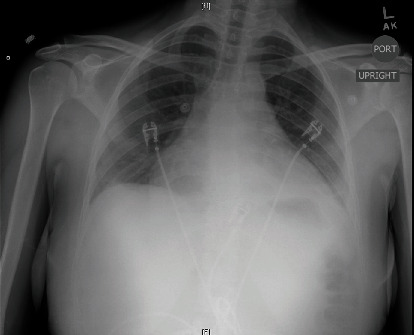
Enlarged cardio-mediastinal silhouette.

A 25-year-old G1P1 female, originally from Brazil, developed worsening dyspnea and pleuritic chest pain immediately postpartum after a normal spontaneous vaginal delivery at an outside hospital. She was diagnosed with acute pericarditis and was successfully treated with an ibuprofen taper. Two months later, she presented to our facility with several weeks of left-sided, sharp, and pleuritic chest pain, coupled with worsening dyspnea, fever, and chills. She was hemodynamically stable and had a heart rate of 120 beat per minute (bpm). Laboratory data were notable for a C-reactive protein (CRP) level of 175 mg/L, white blood cell (WBC) 27.9K/UL, and a TnI of 5.1 ng/mL (Table 1). Electrocardiogram (ECG) showed sinus tachycardia with right bundle branch block (RBBB) (Figure 1), portable chest X-ray showed an enlarged cardio-mediastinal silhouette (Figure 2), and computed tomography (CT) chest with contrast showed a large pericardial effusion without evidence of pulmonary embolism. A subsequent transthoracic echocardiogram (TTE) confirmed the large pericardial effusion with evidence of tamponade physiology. The patient was diagnosed with pericarditis complicated by cardiac tamponade. The pericardial effusion was subsequently drained, and the patient underwent a thorough workup including thyroid testing, HIV, interferon-gamma release assay (IGRA), bacterial and viral cultures, and ANA, which only revealed elevated Coxsackie titers. For her recurrent disease with high-risk features, defined by tamponade and myocardial involvement, she was initiated on a combination of ibuprofen, a short prednisone taper, and 6 months of colchicine therapy.

**Figure 3:**
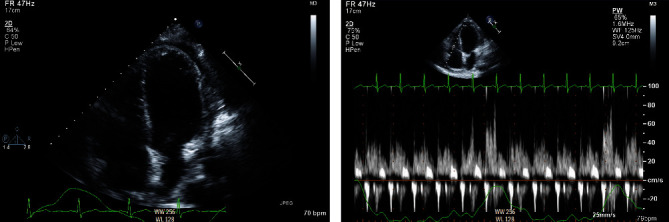
Mild to moderate pericardial effusion with minimal respiratory variation.

Approximately 3 months following, while still on the colchicine, she had another episode of pericarditis, this time with mild to moderate pericardial effusion without tamponade physiology (Figure 3); prednisone was reinitiated.

Within another 2 months, she suffered from another relapse. This was the patient's fourth presentation since her delivery. At that point, the patient underwent another thorough but unrevealing workup including repeat thyroid testing, IGRA, HIV, and ANA in addition to double-stranded DNA, rheumatoid factor, cyclic citrullinated peptide, and genetic studies for familial Mediterranean fever (FMF). Under rheumatology's guidance, a trial of azathioprine starting at 100 mg daily was initiated in concurrence with colchicine and steroids in order to achieve complete remission. The patient was able to slowly come off the corticosteroids, while remaining on colchicine and undergoing active titration of azathioprine to 200 mg.

Over a year from her initial episode, with the disease under control, our patient hoped to conceive again, and she self-discontinued the colchicine. Reassured after several more months of disease quiescence, coupled with fears of teratogenicity, she opted to slowly come off azathioprine. She remained without evidence of recurrent disease for the next year and became pregnant during this time. Although azathioprine was deemed safe by different specialists involved in her care, including her obstetrician, cardiologist, and rheumatologist, the patient opted against taking any maintenance medications during her pregnancy. Therefore, the multidisciplinary team's decision was to treat any flares during pregnancy with corticosteroids.

At 28 weeks of gestation, she presented with shortness of breath, pleuritic chest pain with elevated inflammatory markers (CRP: 120 mg/L) and cardiac biomarkers (TnI of 4.86 ng/mL) consistent with a diagnosis of myopericarditis. She had a mild pericardial effusion on TTE and no evidence of tamponade physiology. She started on prednisone 10 mg daily, and symptoms subsequently subsided with a downtrend of cardiac and inflammatory biomarkers. At that point, prednisone maintenance was initiated at a dose of 10 mg per day planned to be continued up through delivery.

Within 2 months, she presented yet again with a similar clinical picture and was diagnosed with recurrent myopericarditis. Her prednisone was increased to 20 mg daily with symptom resolution. Two weeks later, she went into labor at which pointshe received stress dose steroids and had a normal spontaneous vaginal delivery without any complications. She was discharged on prolonged and slow prednisone taper until her follow-up with rheumatology where the decision was made to reinitiate azathioprine and slowly titrate off the corticosteroids.

## 3. Discussion

Idiopathic recurrent acute pericarditis (IRAP) is quite prevalent, affecting 15-30% patients with a history of acute pericarditis [4]. In women of childbearing age, we aim to optimize maternal and fetal outcomes by planning pregnancy during a time of inactive disease, while also maintaining quiescence throughout the pregnancy. However, both goals come with challenges and are further complicated by the paucity of data surrounding IRAP in pregnancy.

The use of standard first-line agents is limited in pregnancy. Exposure to ASA and NSAIDs after 20 weeks' gestation can lead to premature closure of fetal ductus arteriosus, effectively contraindicating their use in the third trimester [4]. Furthermore, when used in IRAP, it is recommended that these COX-1 inhibitors are added to colchicine. Colchicine is effective in decreasing rates of recurrence of pericarditis [5], and as such, 6 months of tapered colchicine therapy is preferred. As it stands, though, because of its antimitotic mechanism of action, available guidelines do not recommend its routine use in pregnancy [4]. However, a growing body of data suggests that colchicine is probably safe in pregnancy [6], and although there are no well-controlled studies assessing its use for pericarditis specifically, a group of experts in Italy has recently supported its use [3].

Recommendations in pregnant patients continue to include corticosteroid therapy [4], which although effective in the management of symptoms and the resolution of pericarditis, come with their own set of issues. Corticosteroids increase the risk of recurrence [7], making their use somewhat counterintuitive in patients who already suffer from recurrent disease.

For patients like ours, with refractory recurrent disease, family planning is prudent. This label, “refractory” IRAP, is applied to patients who have had at least three relapses and have failed standard therapies [8]. Although data is limited, several immunosuppressive agents have emerged as promising for such patients [1]. Although their use is questionable in pregnancy, these agents can aid in achieving disease quiescence and therefore aid in family planning.

One such therapy is azathioprine, a purine analog, which acts by blocking DNA synthesis and therefore lymphocyte proliferation. Data supporting its use includes a single-center retrospective study of 46 patients with IRAP [9]. These patients were coadministered prednisone, but after 4 weeks, they began tapering off the corticosteroid. Nearly 60% of patients were able to come off both prednisone and azathioprine, while remaining in stable remission. These responders had remained on azathioprine for an average of 14.6 months (Table 2). Although no controlled trials exist regarding its use in pregnancy, data suggests that it is safe, and expert opinion regards it as such (EULAR).

Another therapeutic option in IRAP is intravenous immunoglobulin (IVIG). Data supporting its use comes from a systematic review of case series and reports of refractory recurrent pericarditis, in which just under half of the 30 patients had idiopathic disease [10]. A several day course of IVIG was administered, and this was repeated periodically based on clinical response. A promising 73% of patients were recurrent-free at a follow-up period of nearly three years. While 63% of the patients were initially on corticosteroids, only 17% remained on steroids by the end. With this, IVIG should be considered to induce remission while planning for conception in patients with IRAP. Notably, though, we may also consider its use during pregnancy for IRAP. IVIG is used during pregnancy in a number of disease states and recommended as a first-line option for immune thrombocytopenia in pregnancy [11, 12].

Perhaps most foreign among the immunosuppressive agents to consider are the ilnterleukin-1 inhibitors: anakinra, canakinumab, and rilonacept. Anakinra is a recombinant IL-1 receptor antagonist. Evidence supporting its use in pericarditis includes a small randomized controlled trial of 21 patients with recurrent, colchicine-resistant, and steroid-dependent disease [13]. All 21 patients received anakinra therapy for 60 days, at which point they were randomized to placebo or continued anakinra therapy for the year. Just 2 of the 11 patients who were randomized to receive anakinra had recurrence within the year, versus 9 of the 10 randomized to placebo. Although these data are promising in regard to the therapeutic efficacy of anakinra, they raise the concern of recurrence after discontinuation. This recurrence after anakinra therapy has also been highlighted in a systematic review of published cases [14]. The high rates of recurrence with anakinra may be attributable to its short half-life (4-6 hours), which has led to one group's experimentation with canakinumab, an antibody-based IL-1 antagonist with a half-life of approximately 28 days [15]. The safety profile of these medications in the pregnant women is unknown. However, there is growing data regarding the safety of anakinra and canakinumab in pregnancy [16]. Rilonacept is a made up of the extracellular portions of the interleukon-1 receptor bound to the constant region of an IgG1. A recent phase 3 randomized control trial supports its use in recurrent pericarditis. During an initial run-in period, all patients were treated with a loading dose of rilonacept followed by weekly maintenance dosing. They were then randomized to continue rilonacept therapy or to placebo. 74% of patients (23 of 31) in the placebo group had recurrent pericarditis, while only 7% (2 of 30) of those in the rilonacept arm suffered recurrence. To the best of our knowledge, though, there is no data available to support rilonacept's use in pregnancy, and the FDA labels this agent as pregnancy category C.

## 4. Conclusion

Our patient's experience underscores the necessity of well-controlled trials of therapies for IRAP in pregnancy and highlights the importance of family planning, shared decision-making, and management by an interdisciplinary team comprised of rheumatology, obstetrics/gynecology, and cardiology.

## Figures and Tables

**Table 1:** Cardiac biomarkers and inflammatory markers during disease flare.

1st pregnancy					2nd pregnancy	
Biomarkers	1st (2 weeks postpartum)	2nd (2 months postpartum)	3rd (4 months postpartum)	4th (7 months postpartum)	1st (2 months before delivery)	2nd (2 weeks before delivery)
CRP	175	89.3	41	60.3	45	144
Troponin I	5.1	0.011	0.009	0.006	4.8	5.7

TnI: troponin I; CRP: C-reactive protein.

**Table 2:** Treatment scheme for medical therapy of pericarditis during pregnancy.

Drugs	Postpartum	Postpartum	Pregnancy
	1st episode	Subsequent episodes	
NSAIDs (ibuprofen)	800 mg every 8 hours for 2 weeks	Not used	Not used
Corticosteroids	Not used	Prednisone 10 mg to 20 mg daily with a slow extender taper (6 months)	Prednisone 10 mg to 20 mg based on clinical response with slow taper extender taper (6 months)
Colchicine	Colchicine 0.6 mg twice daily	Colchicine 0.6 mg twice daily	Not used
Azathioprine	Not used	100 mg daily to 200 mg daily	Not used

## Data Availability

None were utilized.

## Abbreviations

CRP: C-reactive protein
TnI: Troponin I
ECG: Electrocardiogram
TTE: Transthoracic echocardiogram.

## References

[1] Imazio M., Brucato A., Rampello S. (2010). Management of pericardial diseases during pregnancy. *Journal of Cardiovascular Medicine*.

[2] Imazio M., Cecchi E., Demichelis B. (2007). Indicators of poor prognosis of acute pericarditis. *Circulation*.

[3] Brucato A., Pluymaekers N., Tombetti E. (2019). Management of idiopathic recurrent pericarditis during pregnancy. *International Journal of Cardiology*.

[4] Adler Y., Charron P., Imazio M. (2015). 2015 ESC guidelines for the diagnosis and management of pericardial diseases. *European Heart Journal*.

[5] Imazio M., Belli R., Brucato A. (2014). Efficacy and safety of colchicine for treatment of multiple recurrences of pericarditis (CORP-2): a multicentre, double-blind, placebo-controlled, randomised trial. *Lancet*.

[6] Indraratna P., Virk S., Gurram D. (2018). Use of colchicine in pregnancy: a systematic review and meta-analysis.. *Rheumatology (Oxford)*.

[7] Lotrionte M., Biondi-Zoccai G., Imazio M. (2010). International collaborative systematic review of controlled clinical trials on pharmacologic treatments for acute pericarditis and its recurrences. *American Heart Journal*.

[8] Imazio M., Lazaros G., Brucato A., Gaita F. (2016). Recurrent pericarditis: new and emerging therapeutic options. *Nature Reviews Cardiology*.

[9] Vianello F., Cinetto F., Cavraro M. (2011). Azathioprine in isolated recurrent pericarditis: a single centre experience. *International Journal of Cardiology*.

[10] Imazio M., Lazaros G., Picardi E. (2016). Intravenous human immunoglobulins for refractory recurrent pericarditis: a systematic review of all published cases. *Journal of Cardiovascular Medicine*.

[11] Abdolmohammadi-Vahid S., Pashazadeh F., Pourmoghaddam Z., Aghebati-Maleki L., Abdollahi-Fard S., Yousefi M. (2019). The effectiveness of IVIG therapy in pregnancy and live birth rate of women with recurrent implantation failure (RIF): a systematic review and meta- analysis. *Journal of Reproductive Immunology*.

[12] Rajasekhar A., Gernsheimer T. (2013). *Clinical Practice Guide on Thrombocytopenia in Pregnancy*.

[13] Brucato A., Imazio M., Gattorno M. (2016). Effect of anakinra on recurrent pericarditis among patients with colchicine resistance and corticosteroid dependence: the AIRTRIP randomized clinical trial. *Journal of the American Medical Association*.

[14] Lazaros G., Imazio M., Brucato A. (2016). Anakinra. *Journal of Cardiovascular Medicine*.

[15] Kougkas N., Fanouriakis A., Papalopoulos I. (2018). Canakinumab for recurrent rheumatic disease associated-pericarditis: a case series with long-term follow-up. *Rheumatology*.

[16] Youngstein T., Hoffmann P., Gül A. (2017). International multi-centre study of pregnancy outcomes with interleukin-1 inhibitors. *Rheumatology*.

